# Impacts of the COVID-19 pandemic on life and learning experiences of indigenous and non-Indigenous university and college students in Ontario, Canada: a qualitative study

**DOI:** 10.1186/s12889-023-15010-5

**Published:** 2023-01-13

**Authors:** Farriss Blaskovits, Imaan Bayoumi, Colleen M. Davison, Autumn Watson, Eva Purkey

**Affiliations:** 1 Department of Family Medicine, Queen’s University, 220 Bagot St, Kingston, Ontario K7L 3G2 Canada; 2 Department of Public Health Sciences, Queen’s University, 62 Fifth Field Company Lane, Kingston, Ontario K7L 3N6 Canada; 3 Indigenous Diabetes Health Circle, Curve Lake First Nation, Kingston, Ontario K0L 1R0 Canada

**Keywords:** COVID-10, University students, College students, Indigenous students, Wellbeing

## Abstract

**Background:**

The years people spend attending university or college are often filled with transition and life change. Younger students often move into their adult identity by working through challenges and encountering new social experiences. These transitions and stresses have been impacted significantly by the COVID-19 pandemic, which has led to dramatic change in the post-secondary experience, particularly in the pandemic’s early months when colleges and universities were closed to in person teaching. The goal of this study was to identify how COVID-19 has specifically impacted the postsecondary student population in Kingston, Ontario, Canada.

**Methods:**

The Cost of COVID is a mixed methods study exploring the social and emotional impacts of the COVID-19 pandemic, with a focus on families, youth, and urban Indigenous People. The present analysis was completed using a subset of qualitative data including Spryng.io micronarrative stories from students in college and university, as well as in-depth interviews from service providers providing services to students. A double-coded phenomenological approach was used to collect and analyze data to explore and identify themes expressed by postsecondary students and service providers who worked with postsecondary students.

**Results:**

Twenty-six micronarratives and seven in-depth interviews were identified that were specifically relevant to the post-secondary student experience. From this data, five prominent themes arose. Impacts of the COVID-19 pandemic on the use of technology was important to the post secondary experience. The pandemic has substantial educational impact on students, in what they chose to learn, how it was taught, and experiences to which they were exposed. Health and wellbeing, physical, psychological and emotional, were impacted. Significant impacts were felt on family, community, and connectedness aspects. Finally, the pandemic had important financial impacts on students which affected their learning and their experience of the pandemic. Impacts did differ for Indigenous students, with many of the traditional cultural supports and benefits of spaces of higher education no longer being available.

**Conclusion:**

Our study highlights important impacts of the pandemic on students of higher education that may have significant individual and societal implications going forward. Both postsecondary institutions and society at large need to attend to these impacts, in order to preserve the wellbeing of graduates, the Canadian labor market, and to ensure that the pandemic does not further exacerbate existing inequalities in post-secondary education in Canada.

## Background

University and college years are often full of formative experiences when young people explore interests, socialize, and begin to develop their adult identity. Students may begin to develop an understanding of what is important to them, who is important to them, and what they want to do with their lives through assessment of their own values as well as those of close relationships and social groups [1]. Identity and intimacy overlap when young adults both develop their sense of self and use social interactions to clarify personal values, gauge successes and expectations [1, 2]. This development creates distance from previous family or social roles, and students start to navigate social interactions and extracurricular activities independently. Doing so develops critical thinking skills, teamwork, and can enable higher levels of academic and future professional performance [3, 4].

Changes and stresses naturally occur during this life stage and time of transition. Establishing identity and career goals is one such change. In addition to scholastic and identity formation, there are also practical concerns of coordinating housing, cooking, finances, and work-life balance which most students must manage for the first time [5].

Unique challenges may be present for students from equity-seeking groups. Rheinschmidt and Mendoza-Denton found that students of higher education from lower earning households worry about unfamiliar cultural norms or stereotypes, possibly leading to anxiety-provoking expectations of discrimination which can impact school performance [6]. This discrimination can be subtle, such as the experience of feeling ‘othered’, enduring microaggressions, or experiencing a sense of hypervisibility when speaking out against colonialism and racism [7]. Alternatively, it may be explicit, such as in Currie et al. where Indigenous postsecondary students faced deliberate housing discrimination [8]. Literature involving postsecondary and graduate Indigenous students shows that academic success often hinges on a sense of community provided by Indigenous peer and faculty support [9, 10]. Self-compassion and institutional supports can help students navigate stressors, especially for women, sexual minorities, and first-generation postgraduate students [11].

Before the COVID-19 pandemic, Indigenous women in Canada were half as likely to hold a bachelors degree as non-Indigenous women (14% vs 32%), and Indigenous men were less than a third as likely to hold a bachelors degree as non-Indigenous men (8% vs 27%) [12]. Despite the gap, these numbers represent a significant increase in Indigenous achievement in higher education between 2006 and 2016.

The COVID-19 pandemic imposed many essential restrictions on individuals and institutions. Schools had to accommodate quickly to changing public health requirements. This meant that most classrooms became virtual, limits were imposed on group learning [13], and professional development opportunities declined – especially in degrees requiring practical hands-on training [14, 15]. Frequent changes to work schedules, school schedules, access to resources, and program expectations contributed to added stress. The need for technology in this new environment was financially limiting for some more than others [16]. For example, many Indigenous students from remote communities encountered further challenges with irregular access to computers or limited ability to connect via high-speed internet [17]. The impact of remote learning differed among students, with students who had previously attained lower grades being disproportionately challenged [18].

Typical coping mechanisms for the pre-pandemic stressors such as physical activity, social interactions and student meetings, were limited at the beginning of the COVID-19 pandemic [19, 20]. This coincided with decreased mentorship and institutional support which created an environment that challenged success [20].

The present analysis seeks to explore the experiences of post secondary students during the first 9 months of the pandemic in a medium sized Canadian city. This study is unique in that it includes experiences of both students and post-secondary service providers, and intentionally includes the voices of Indigenous students and their service providers. As mentioned above, there remain important disparities between Indigenous and non-Indigenous Canadians in higher education, and ensuring that recent gains are not lost due to changes related to the COVID-19 pandemic remains important in any analysis of the impact of the pandemic on higher education in Canada.

## Methods

### Setting

The data for this analysis were drawn from a large mixed-methods study entitled the Cost of COVID, which explored the social and emotional impacts of the COVID-19 pandemic in Kingston, Frontenac, Lennox, and Addington public health region (KFL&A) of Southeastern Ontario, Canada as reported in previous manuscripts [21–23]. The overall study collected data from across the community, but with an intentional focus on families and youth, as well as on collecting information on the experiences of urban Indigenous People and people living in poverty, who face barriers to participating in research and whose voices are often missed in such projects. Data were collected from June 2020 until November 2020. KFL&A is an area with a population of 210,000 people that was relatively spared during the first year of the pandemic, with 758 cases of COVID-19 and a single death as of the end of the study period. The region had a strong public health response, with and excellent communication strategies, strong leadership, and high levels of compliance with public health guidelines [24, 25]. There are three institutions of higher education in Kingston: Queen’s University, St Lawrence College, and the Royal Military College of Canada. During the study period, all these institutions conducted remote learning for the vast majority of their students.

Ethics approval for the study was obtained through Queen’s University Health Sciences and Affiliated Teaching Hospital’s Research Ethics Board. An Indigenous oversight committee was created in collaboration with the Indigenous Health Council (IHC) to ensure that any Indigenous data collected was in alignment with the First Nations principles of Ownership, Control, Access and Possession (OCAP®). Specifically, the oversight committee was involved in research question approval and helped guide interpretation of results as they pertained to Indigenous people [26].

### Data sources

This analysis includes data from two sources:

- *Spryng.io Micronarratives:* Spryng.io is an online data collection platform that allows for rapid collection of a large number of short stories or “micronarratives”. Inclusion criteria were adults over 18 years of age consenting to the study, and participants were recruited using a combination of convenience sampling (via email, social media, flyers and posters) and purposive sampling (at sites and events frequented by target populations, including Indigenous People). Participants were asked to tell a short story in response to one of three study prompts: (1) *Please tell a story about the worst OR best impact of COVID-19 on you and/or your household;* (2) *Please tell a story about how the COVID pandemic has affected you physically, mentally, spiritually or emotionally;* or (3) *Fast forward a year from now. What memorable story would you tell a friend about how COVID-19 affected you and/or your household?* This story could be dictated and automatically transcribed through online software, or typed directly by participants. Stories ranged from 150 to 800 words, with most falling between 250 and 500 words. Some demographic information was collected, however names and uniquely identifiable information were not.
- *In-depth Interviews:* The in-depth interviews used for the Cost of COVID Study have been described elsewhere [21–23]. These were conducted with key informants recruited from organizations providing healthcare and social services in KFL&A, including service providers in child welfare services, school support, mental health services, and domestic violence services, among others. Intentional recruitment was performed of service providers from organizations providing services to Indigenous Peoples. Service providers were interviewed using a semi-structured interview guide to facilitate reporting on changes observed in their clients throughout the COVID-19 pandemic. Interviews were completed via Zoom, between October 2020 and November 2020. This video conferencing platformwas used in order adhere to public health social distancing guidelines. Interviews were audio recorded through the Zoom platform, and transcribed in their entirety.

### Participant selection

For the purposes of this analysis, the Spryng.io micronarratives relevant to the college and university experience were identified using a set of keyword searches in the full data set. Keywords included: course, degree, grad*, school, stud*, college, university, class*, Queen*, St. Lawrence, Lawrence*. Of 211 micronarratives collected, 26 were retained for analysis, including 4 from self-identified Indigenous students. In depth interviews relevant to the college and university experience were identified based on the job descriptions identified by participants (eg: student advisor). Of the 32 collected, 7 were retained for analysis, including 3 from Indigenous service providers. Our total sample for data analysis therefore involved 33 transcripts.

### Data analysis

A phenomenological approach was used to understand themes expressed by postsecondary students and service providers who worked with postsecondary students [27]. Phenomenology is the study of people’s lived experience of a phenomenon, in this case the postsecondary student experience during the COVID-19 pandemic, and provides an up-close and first-person perspective on a complex topic or phenomenon [27]. Data transcripts were read in their entirety by two researchers (FB and EP), and inductive coding was performed using NVIVO software. Initial themes were progressively compressed through thematic groupings to move from over 30 themes to 15. Through co-analysis both researchers further narrowed themes until 5 main themes were identified.

## Results

As discussed, 211 micronarratives Spryng.io micronarratives were collected for the Cost of COVID Study. Of these, 26 were retained for this analysis using key word search, including 4 from self-identified Indigenous students. Thirty-two service providers interviews were conducted for the larger study, and 7 were retained as relevant to postsecondary students, including 3 from Indigenous service providers.

The prominent themes that arose from our phenomenological analysis were impacts of the COVID-19 pandemic on the use of technology, on education, on health and wellbeing, on family, and on finances.

### Impact on the use of technology

Participants described the expanded use of technology, that is any use of hardware or software, as core for schooling during the COVID-19 pandemic. Technology has been a necessary aspect of university and college life for many years, but the pandemic has forced technology to become the focus of educational efforts. Participants noted that exams and classes now needed to be taken online rather than in-person. Students needed to locate and adjust to virtual research and volunteer opportunities as well. Postsecondary institutions were finding ways to limit disruptions to learning and individuals’ degrees as much as possible by switching formats, but the impact on the students in our study was substantial. Responses were mixed regarding how this reliance on technology affected students on an individual level. Some respondents found that this was a positive change in their lives. One individual with chronic health concerns found the lack of commute – now having the ability to work from home – easier on their health. And another participant found that it allowed for more time to spend with family:

“I’m considered at risk of being immune compromised because of my condition. […] I’m doing online learning there’s positives to it […] that I don’t have to leave and go through the whole process of travelling.” (Spryng.io, 5638).

“I will focus on the best impact which would of course be the extra time our family has been able to share while working and attending school from home. My significant other started a new job and I began a new semester in university, and having the extra time during these busy schedules to still see each other through it has been extremely nice and brought us even closer together.” (Spryng.io, 5626).

Others, however, found the new integration of specific technologies into their lives dehumanizing and detrimental. They became fatigued with screens and technology, and any support offered remotely either felt like more work, or there was not enough of a personal connection to truly help. Ironically, through increased virtual connection and entering people’s homes remotely, there was a related decrease in emotional connection and support – and at times an invasion of privacy. Students noted that it was convenient to work from home, but social isolation was challenging, even if family members were nearby. A service provider noted that they would typically be able to read body language and tell if someone needed to talk if they were in-person, or they could leave a door unlocked for office hours to allow students to come in of their own volition which was no longer possible:

“I find it hard to continue living life without the small things like going to the movies, bars and spending time with large groups of friends. I find that FaceTime calls and zoom meetings do not even begin to compare to the human interaction we once had.” (Spryng.io, 5946).

“Typically in an in-class capacity, you know, I can see students. I can see if they’re triggered by something. You get to see these emotional reactions and if someone needs to get up and leave because of a conversation, you know to follow-up with them. And we don’t have that benefit in a virtual context because they leave their cameras off. So you never have any idea.” (Service Provider, 10A).

“You know, it’s weird because there’s again a bit of oxymoron between, like a total lack of privacy and being lonely. And I think like that’s interesting for all of us that like we’re in each other’s houses. And a lot of them have nowhere to go and so I’ll be in their bedroom, right. Like they’re sitting in their bedroom on their computer and I can see their unmade bed.” (Service Provider, 6A).

The switch towards remote learning had both positive and negative outcomes. One service provider highlighted this well, recognizing the role that technology played for the rural student population at a local postsecondary institution. In some cases, having a virtual option broke down barriers, but on the other hand some students (particularly Indigenous respondents) had limited access to internet and technology such as laptops and found face-to-face learning more beneficial to the academic experience:

“Even when we go back, you know if things return to somewhat normal, it is nice to have that virtual option for students who maybe don’t have transportation and live in a rural area outside of our area, to be able to participate in after-hours events with other students and be able to participate in some of those conversations” (Service Provider, 10A).

“I think it’s affected Indigenous people, especially the ones that don’t have access to technology, that don’t have internet. Because like so many people in our community do not have access to internet. Like they would, like to get internet they would go sit like outside a Tim Hortons or a library or somewhere. And when those were all locked down, then it was harder for them to access” (Service Provider, 12A).

“And what students are looking for is this ability to connect through, you know, feasts and crafts and things that they can kind of put their hands on and take them away from their study. And in this virtual capacity, they don’t want one more screen time in a day of screen time.” (Service Provider, 10A).

### Impact on education

In this study, the “education” theme encompassed student performance, delivery of academic materials and opportunities, access to professors and tutors, and the overall academic structure. The pandemic disrupted the education process in numerous ways including school year being cut short, lack of celebration of successes, and altered opportunities as programs shifted towards remote classes and learning. These disruptions were compounded with the other themes uncovered in our results creating challenges for students and institutions alike.

The pandemic forced individuals to relocate, with numerous respondents moving back to their family homes more than a month before they had intended to. Some had to move from out-of-country, and others moved home when they were intended to remain at school for summer courses. While these major changes were occurring, some students felt that their schools were not supporting them:

“The worst impact of the pandemic on me was having to leave my studies and long term my relationship in California to come back home to Canada.” (Spryng.io, 5947).

“My job and volunteer roles were all ended, and I was forced to try and complete a thesis and 3 courses at my parents’ home. […] I feel [name of University] has made VERY little effort to celebrate their graduates, especially compared to other universities and high schools.” (Spryng.io, 4051).

Staying home meant, for some respondents, that they now needed to balance home life with their school work. This, in some situations, meant they had more time for school work, whereas in other cases home responsibilities took over:

“Before the pandemic I would have had to work [outside of the home] three to four times a week and I believe it was impacting my grades to some degree.” (Spryng.io, 5631).

“Worried about the effects on my schooling if they [respondent’s children] don’t return to school full time themselves in the fall.” (Spryng.io, 4095).

There were also effects on students’ education directly. Students were not able to relate to material in the same way and isolating at home also limited access to tutoring and support services which further impacted success. Some also worried about the lack of exposure to professional development and volunteer opportunities. One service provider also highlighted the challenge of providing support virtually, which has led to changing degrees and academic concerns:

“We did see a little bit of a drop off of students who withdrew after the mid point in the first semester. So we had some movement of course either within different programs to switch to programs that might be more conducive to like a virtual learning environment versus some of the hands-on programs that they might have had previously. So switching to like general arts versus, you know, nursing or something that maybe more challenge in a virtual context. So there’s been that movement as well with students changing their studies. And with us not being able to outreach or connect with them on a regular basis, we’re anticipating that there will be, you know, concerns at the end of the semester of students who um didn’t complete their semester or somehow kind of got lost in the shuffle of life, and fallen behind.” (Service Provider, 10A).

Despite these worries, there was still hope and acknowledgement that this new virtual world opened opportunities to students that wouldn’t have been considered previously. There was a positivity in these responses, a hopeful surprise that new opportunities became available in a stressful time:

“Suddenly, no one was able to access electives and we had new opportunities to do online research and home electives in specialties of medicine that we didn’t have before. So, initially seemed like a big shock factor for applications, turned out to be a blessing in disguise through which we could do better career exploration.” (Spryng.io, 4133).

“She was able to find a couple of Professors to get hours with and in doing so landed a research assistant position. Getting paid and being given credit as co-author in a publication she would not have known about had she been in Italy.” (Spryng.io, 4122).

### Impact on health and wellness

Many participants discussed the impact of the pandemic on their health and wellbeing. These responses involved negative and positive impacts on mental, physical, and emotional wellbeing, including health and safety. Participants discussed safety, or lack thereof, as any threat impacting holistic wellbeing, including within the household, due to a specific stressor, or a more generalized threat.

Mental health was a common sub-theme among respondents. Anxiety and depression due to social isolation was commonly mentioned to be an issue for postsecondary students:

“Like a really huge increase in the amount of anxiety that like people coming to me saying that they feel anxious and they’re having panic attacks or that they feel like really depressed because of the situation” (Service Provider, 12A).

“I am so lonely and sad and feel like I have no purpose. This has been one of the worst experiences of my life and I have never felt so alone and isolated […] I struggled with an eating disorder in high school, and being home all day with nothing to do but make food, and the gym being closed has made me gain weight” (Spryng.io, 4051).

Some respondents also identified changes in substance use:

“I think during COVID, you’re just by yourself and no one knows what you are doing… I remember helping one student who was living in a house where everyone was using. And they said, I just can’t live here. We had to try and find a different accommodation for that person.” (Service Provider, 12A).

Not all references to mental health during the pandemic, however, were negative. Some discussed trying to stay positive and looking for the best in their present situations:

“I intend to enjoy self-isolation by appreciating what I can do and at least attempting to learn some skills that so far have evaded me.” (Spryng.io, 5561).

Some respondents felt that the impacts of isolation and Public Health restrictions affected people disproportionately, such as certain Indigenous People experiencing fractured communities, and decreased support:

“We have felt constant pressure from our peers to on one hand bend the rules, make exceptions; and on the other hand, uphold the precautions of public health experts, “do the right thing“, even as we watch our community grow more distant and fragmented.” (Spryng.io, 6011).

While not unique to postsecondary students, the necessity of isolation also made emotional abuse and controlling behavior potentially more feasible and even more hidden that usual among this population as well. Decreased public supports due to daycare closures and decreased work hours meant that some students need to rely on, or more frequently interact with, abusive family members who could previously be kept at a distance. A service provider further recognized the threat to physical violence that has occurred during the pandemic as well:

“My husband’s covert narcissism was enabled by the social isolation required to survive the pandemic. It really made me feel even more trapped and enraged […]”. (Spryng.io, 5791).

“And the reality is that there are people who have things going on at home, such as domestic violence or family conflict with other people in their home” (Service Provider, 10A).

Physical health was also discussed, with a mixture of negative and positive associations. Some individuals found that a lack of routine and overall stress contributed to negative physical health, whereas others found that their new routines allowed for more time to improve their overall health. Physical health was impacted for some respondents due to how people around them chose to ignore Public Health recommendations. It was an emotional threat with the implications for physical health and understanding that certain individuals were not concerned with the safety of those around them:

“I was better able to spend a lot of time rebuilding both my physical and mental health”. (Spryng.io, 4157).

“Isolated as I live at home but still do not feel comfortable living in the ghetto with the other students who can’t take the pandemic seriously”. (Spryng.io, 5404).

### Impact on family

Participants discussed family both as the nuclear or extended family in the Western sense, as well as those identified as chosen family by the respondent. This could include friends, teachers, community members, and others the respondents felt connected to. Individuals noted both positives and challenges regarding the impact of the pandemic on family and community interactions.

Some respondents found that working from home or moving home earlier than expected impacted work-life balance. And one service provider highlighted how this could disproportionately affect Indigenous learners:

“I’m a single mother with three kids ten and under, I am also a full-time distance Ed university student. As a result of school closures, I have had my children with me for my whole semester”. (Spryng.io, 4095).

“A little bit differently in the sense that some of our Indigenous students are also caretakers for siblings and things, you know, [for] their parents. Have taken the siblings from the home and they’ve moved in with them. So it’s a little bit different of a dynamic and so they’re also juggling those caregiving responsibilities as a student for a child that, you know, belongs to the family but is not necessarily their own.” (Service Provider, 10A).

It was clear how important relationships and family were to students, with changes in relationships and family dynamics. Some individuals needed to move home to complete their school year, and despite being close to family felt alone because of different schedules. Others found that they worried about the virus and the impact on different family members. And in other instances, the pandemic was able to mend families and connect them in new ways over distances:

“We played games on the weekend, walked together most evenings and enjoyed creating an oasis on our back yard for the summer. […] It was a time we will always remember getting through together”. (Spryng.io, 4118).

“My desire to heal and recover from the dysfunction in my family and marriage was stymied”. (Spryng.io, 5791).

Relationships with community were also referenced. Community was an important contributor to well-being. Indigenous service providers highlighted the loss of community in the post-secondary context:

“We had to stop offering certain services. Normally we have a lounge, which doesn’t sound like a big deal but it actually really is. It’s an Indigenous space. We have medicines. It is the only space on campus other than productions where the students can smudge when they need, no questions asked. It’s sad that we can’t offer that to them because I think a lot of them who are traditional people need that. And also we have often a number of students who are Indigenous but who don’t have culture. And so having access to that space is a doorway into understanding how the culture is or can be in their life.” (Service provider, 6A).

“Our community has been cracked open and there now exists a deep divide between those who live in the house and the rest of our members”. (Spryng.io, 6011).

### Impact on financial and socioeconomic situation

The pandemic led to altered financial situations for multiple respondents. Many businesses and institutions were forced to close in accordance with local Public Health restrictions to prevent spread of illness. Consequently, students lost their jobs due to business closures, or lost work hours due to infectious symptoms or needing to care for their families due to school or activity closures. In direct response, some students also found themselves changing socio-economic or income brackets, with particular impact on mature students with families to support at home:

“I am now in a low income situation so it’s not like I can go online and get counselling that way because that cost[s] money that are not within my means” (Spryng.io, 5638).

“With 3 kids all in school and with only one source of income, everything is on [a] tight budget” (Spryng.io, 5823).

A service provider recognized the challenge to students and the lack of part-time employment opportunities. Losing access to part-time jobs impacted access to benefits cutting off Employee Assistance Programs and other supports. One student chose a lower paying job for experience rather than accept CERB (Canada Emergency Response Benefit), a government program for unemployed individuals during the pandemic:

“Because of COVID and a lot of people lost their part-time jobs even, you know, our students who typically would have been working over those summer months to gain that little bit of extra income, lost that opportunity as well.” (Service Provider, 10A).

“[When] CERB came out for students she would rather this job opportunity then that money because the experience is invaluable.” (Spryng.io, 3895).

At least one student needed to physically move and find a student to lease their apartment. Another individual had chronic health concerns, and in addition to the threat against health, there was the added threat of losing their home during the pandemic given an inability to work. There was also inadequate pest control in certain houses, and some concerns about the housing conditions contributing to worsening health outcomes as well:

“it’s affected my living situation I live in [low] income housing and it seems that any services that existed, basic maintenance was a, it’s less than up to par the one thing that I’ve noticed this year is there is more of everything: bugs, rats anything in the animal kingdom seems to be over populating in my area.” (Spryng.io, 5638).

One service provider highlighted the challenge of completing an online education while enduring poverty. Individuals come to school expecting to pay a great deal of money for tuition, but that typically the trade-off would be taking classes, good education, new community, and an ability to supplement income with work and tutoring. COVID-19 has, at times, completely cut off these positives:

“I think for like people living in poverty don’t necessarily have access to internet and like when everything was over the internet, they’re not going to get that.” (Service Provider, 12A).

“Poverty is, people who wouldn’t have been, who wouldn’t have thought themselves poor before are struggling more because of like added bills and rent and apartments that they don’t need to be renting. That’s a big one. People who moved here thinking that they were going to be in class, and they rented an apartment at astronomical [local] rates. And now they’re paying for this apartment and not working. And it wouldn’t have felt so bad if they if they could have been, like if they were here and in class, then you just kind of live with it. But now they could be living much more cheaply in their home community and not struggling with that. So that’s hard.” (Service Provider, 6A).

## Discussion

The COVID pandemic has impacted postgraduate students in substantial and varied ways, both positive and negative, including changes related to the use of technology, the topography of respondents’ education, general wellbeing including mental and physical health, interactions with family and friends, and finances. Many of these themes overlapped and impacted one another. While the landscape of postsecondary education has changed substantially since the early months of the pandemic, three areas warrant further discussion. These areas have potential impacts on long term well being, and can therefore influence policy and programming during the recovery from the COVID-19 pandemic.

The first is the impact of the COVID-19 pandemic on identity formation and development among college and university students. University and college changed from being a time of connecting, development and learning from a likeminded community, to one in which students needed to rely on themselves alone to overcome a myriad of new obstacles, in the context of social isolation and decreased institutional supports [19, 20]. Resilience can arise from community-belonging, typically in a shared physical and emotional space, an ability to support one another and bolster each other’s learning and growth [28]. Some students were able to increase the connections they made locally, often forced to do so in contexts where they would otherwise have travelled (for elective experiences, work study, etc) but could not due to the pandemic. Service providers and institutions tried to continue the tradition of community building and belonging, but virtual gatherings were a weak replacement, as many students had too much screen fatigue to access virtual supports, designed to help bolster wellness [3, 29]. The lack of robust identity formation, in collaboration with others, may have long term implications for young adults’ well-being and functioning in society.

A second area, directly related to the first, is the impact of the changes to higher education on long term outcomes such as matriculation and employment, with all the consequent individual socioeconomic and community economic implications thereof. The increased stress and decreased mental health illustrated by our findings, as well as decisions to change programs of study due to the logistics of online learning are important not only because they affect student wellbeing and academic development, but also because they can directly impact student and institution success via decreased matriculation rates and changes in career and employment choices and opportunities which may carry on far into the future [30]. Long term impacts include changes in the labor market, with fewer people trained in certain key sectors. Pre-existing gaps in academic achievement between Indigenous and non-Indigenous students may be widened if specific attention is not paid to differential impacts of the pandemic. Additionally, student poverty or limited funds, typically common in up to a third of postsecondary students [31], seems to have become more of a concern during the pandemic with limited job availabilities and the need for expensive technology and good internet connections. Focusing on survival does not allow students the opportunity to perform academically, and this widening of socio-economic disparity may increase the gap in academic success and wellbeing between groups of students and have implications for long term, broader socioeconomic inequality. On the other hand, however, increased access to remote education improved accessibility for certain subgroups. Some of our participants indicated that accessibility was improved for those with chronic health conditions, as well as for students from rural or remote areas, provided that internet access was affordable and available. This illustrates the importance of having multiple options, and different ways for students to access education and extracurricular learning experiences. While fully virtual educational opportunities are one method, moving forward it may be possible to meaningfully organize hybrid learning opportunities to give learners increased choice in how they access education.

Finally, in addition to differential impacts on students experiencing socioeconomic disadvantage, the pandemic changes to higher education differentially impacted other traditionally marginalized student groups as well. Our own findings indicate that during the study period, sense of isolation related to the COVID-19 pandemic was pronounced in Indigenous individuals in the Kingston area. Evidence from post-secondary and high schools has found that positioning a caring and supportive individual in a role to help students can be beneficial [32] and having Indigenous-led peer-support can contribute to a sense of belonging [33]. Access to community members, Elders, tutors, and traditional ceremony and practices contribute to academic success of minority student populations [34]. The resources that have been created in local colleges and universities to support Indigenous students’ wellness, therefore, including drumming, song, introduction to traditional medicines, smudging, and access to student lounges and computers, are essential services, not luxuries. It is imperative to find meaningful ways to transition that support to alternative platforms because many have been and continue to be inaccessible or un-accessed during COVID-19 due to a lack of in-person activities. This will be important to ensure that pre-pandemic gains in the academic achievements of Indigenous students are not lost due to the decrease and changes in these services. Indigenous service providers creatively used virtual group meetings, beading circles, and mailing materials to students to help bolster this gap; nevertheless, students described a striking loss of connection. Students and providers lost access to informal means of help-seeking and opportunities to assess and support student wellbeing. A switch to a mixed or blended platform with small in-person and virtual supports may be needed and is consistent with mixed reports of harm and benefit in our study. These have been beneficial in other settings [35, 36].

## Limitations

This analysis presents several limitations. The cost of COVID-19 Study was designed to capture multiple experiences, not purely those associated with higher education, and our sampling would have been slightly different had our study been specifically targeted towards this topic. This would have resulted in a larger number of micronarratives, as well as a more diverse group of service providers in in-depth interviews. Additionally, our data is confined to the first stage of the pandemic, ending in January 2021, and given the ongoing restrictions posed on higher education for many months after this, some experiences that took longer to materialize may have been missed. Nevertheless, our data is sufficient to illustrate the important impacts of changes to higher education that occurred at the beginning of the pandemic, consistent with literature emerging from Ontario and elsewhere [37].

## Conclusion

Our study highlighted the compression of students’ worlds, academic and non-academic, throughout the COVID pandemic, and the impact this had on technology, education, wellbeing, family, and finances. These findings can contribute to programming and policy directions as we emerge and evolve within the COVID-19 pandemic. Supporting students and young adults in identity formation and community connectedness can be an intentional goal of institutions of higher education. Ensuring that shifts in career paths stemming from online learning are rectified, when appropriate, to respond to student desires and to the Canadian job market, will be important. Attending to the differential socioeconomic implications of the pandemic on students from different income brackets through the provision of bursaries and support for higher education can limit the impact of the pandemic on social inequality. Finally, identifying the differential needs of certain groups of students is key to ensuring the ongoing success of all students, including specifically Indigenous students, as well as those from racialized backgrounds or lower socioeconomic brackets, in order to ensure that graduates from institutes of higher education reflect the diversity and strength of Canadian society.

## Data Availability

Deidentified study material is available upon request from authors. Given the sensitivity of the qualitative transcripts, they will not be made available.
